# TiO_2_-Nanobelt-Enhanced, Phosphorescent, Organic Light-Emitting Diodes

**DOI:** 10.3390/nano15030199

**Published:** 2025-01-27

**Authors:** Sushanta Lenka, Shivam Gupta, Bushra Rehman, Deepak Kumar Dubey, Hsuan-Min Wang, Ankit Sharma, Jayachandran Jayakumar, Ching-Wu Wang, Nyan-Hwa Tai, Saulius Grigalevicius, Jwo-Huei Jou

**Affiliations:** 1 Department of Materials Science and Engineering, National Tsing Hua University, 101, Sec. 2, Kuang-Fu Road, Hsinchu 30013, Taiwan; sushantalenka1@gmail.com (S.L.); shivam19gupta@gmail.com (S.G.); bushra@gapp.nthu.edu.tw (B.R.); peter900621@gmail.com (H.-M.W.); int.ankit96@gmail.com (A.S.); jayakumar@mx.nthu.edu.tw (J.J.); nhtai@mx.nthu.edu.tw (N.-H.T.); 2 Advance Research, First Solar Inc., Perrysburg, OH 43551, USA; deepakkumar.dubey@firstsolar.com; 3 Graduate Institute of Optoelectronics, Department of Mechanical Engineering, National Chung Cheng University, Chiayi 62102, Taiwan; melcww@ccu.edu.tw; 4 Department of Polymer Chemistry and Technology, Kaunas University of Technology, Radvilenu Plentas 19, LT50254 Kaunas, Lithuania

**Keywords:** TiO_2_ nanobelts, hybrid hole-injection layer, doping, OLED

## Abstract

This study investigates the enhancement of organic light-emitting diode (OLED) performance through the integration of titanium dioxide (TiO_2_) nanocomposites within a poly(3,4-ethylenedioxythiophene) polystyrene sulfonate (PEDOT/PSS) matrix. The nanocomposite films were prepared using a controlled dispersion of TiO_2_ belts into the PEDOT/PSS solution, followed by their incorporation into the OLED hole-injection layer (HIL). Our results demonstrate a significant improvement in device efficiency, attributed to the optimized charge carrier mobility and reduced recombination losses, which were achieved by the presence of TiO_2_. The nanocomposite hybrid layer enhances light emission efficiency due to its role in modifying surface roughness, promoting better film uniformity, and improving hole injection. The incorporation of TiO_2_ nanobelts into PEDOT/PSS led to significant efficiency enhancements, yielding a 39% increase in PE_max_, a 37% improvement in CE_max_, and a remarkable 72% rise in EQE_max_ compared to the undoped counterpart. This research provides insight into the potential of TiO_2_ nanocomposites in advancing OLED technology for next-generation display and lighting applications.

## 1. Introduction

Over the past few decades, organic light-emitting diodes (OLEDs) have garnered substantial interest due to a multitude of advantages, making them a prime focus in display and lighting technologies [1,2]. Key attributes of OLEDs include a wide viewing angle, enabling enhanced visual clarity from multiple perspectives, and exceptional mechanical flexibility, allowing for innovative designs such as bendable and foldable screens [3,4,5]. Additionally, OLEDs operate at low driving voltages, which not only improves energy efficiency but also extends device longevity, while their relatively low manufacturing costs make them economically appealing for large-scale production [6,7]. To broaden the reach and application of OLED technology in diverse markets, continual progress in theoretical understanding, device structural optimization, and advanced material innovations is essential. A critical aspect influencing OLED efficiency and longevity lies in the interface characteristics between the electrode and the organic layers [8,9]. The performance of OLEDs is highly dependent on minimizing the energy barrier at this interface, as it plays a significant role in charge injection and transport. Consequently, careful selection of electrode materials is imperative, as they directly impact the overall device efficiency, operational stability, and potential for high-performance applications across consumer electronics, automotive displays, and ambient lighting [10,11].

The operational principles of OLEDs are based on the diode mechanism. Each layer within the device operates according to a distinct mechanism. To improve device performance, researchers are focusing on optimizing various layers. Specifically, when considering the hole-injection layer (HIL), the efficiency of the OLED depends on the effective injection of charge carriers and their transport to subsequent layers within the device structure [12,13]. The efficiency of charge injection and transport is significantly enhanced by mechanisms such as quantum tunneling, which enables charge carriers to overcome potential barriers that might otherwise hinder their flow [14,15]. Additionally, at material interfaces, misalignments in electronic states can form interface dipoles, creating localized electric fields that direct charge carriers to the appropriate layers, thereby improving injection efficiency. Optimizing these mechanisms, especially the functionality of the HIL, is critical for enhancing the overall efficiency and reliability of OLEDs. Among conducting polymers, poly(3,4-ethylenedioxythiophene) polystyrene sulfonate (PEDOT/PSS) has emerged as a versatile material for electronic applications, including OLEDs, organic solar cells, organic thin-film transistors, and electrochromic devices. PEDOT/PSS thin films are particularly notable for their high transparency and conductivity, with typical values around 10 S/cm [16]. These films are fabricated using techniques like spin-coating, which produce stable layers under ambient conditions, further supporting their integration into electronic devices. The unique properties of PEDOT/PSS make it an excellent candidate as a charge-transporting or charge-injecting layer in OLED architectures. The electrical, optical, and magnetic properties of PEDOT/PSS can be fine-tuned through the incorporation of inorganic materials [17,18]. The emerging field of dopant engineering in metal oxides has played a pivotal role in advancing materials that enhance OLED performance. Metal oxides such as titanium dioxide (TiO_2_), tin dioxide (SnO_2_), barium stannate (BaSnO_3_), tungsten trioxide (WO_3_), indium oxide (In_2_O_3_), and zinc oxide (ZnO) have been extensively studied as electron transport layers (ETLs) in electronic systems [19]. These materials offer superior electronic properties, including high electron mobility, wide bandgaps, and excellent stability, making them highly effective for improving device efficiency. Recent studies have also investigated the potential of doped metal oxides as HILs to further optimize device performance [20,21]. Doping enhances the conductivity and optical properties of these materials, leading to improved charge carrier balance and energy level alignment within the device. Among the metal oxides, titanium dioxide (TiO_2_), particularly in its anatase phase, has shown remarkable hole migration properties [22]. Both the anatase and rutile phases of TiO_2_ influence charge transport and recombination dynamics, largely due to the behavior of hole polarons within their structures [23,24]. The anatase phase is especially noteworthy for its superior hole mobility, attributed to its lower effective charge carrier mass and reduced recombination rates compared to the rutile phase [25,26]. Additives such as zinc oxide (ZnO), titanium oxide (TiO_2_), cadmium sulfide (CdS), cadmium selenide (CdSe), and various metal nanostructures have demonstrated their effectiveness in enhancing the functionality of conducting polymers like PEDOT/PSS [27,28,29,30]. For instance, the integration of TiO_2_ with PEDOT/PSS exploits TiO_2_’s wide bandgap of 3.37 eV, a large exciton binding energy of 60 meV, high chemical stability, and outstanding electrical and optical properties. Recent studies have highlighted the versatility of TiO_2_ metal oxide materials in advanced electronic and photoelectrochemical applications. Xavier et al. demonstrated the use of TiO_2_ as an efficient hole transport layer for photoelectrochemical systems [31]. Gupta et al. explored the incorporation of TiO_2_ nanoparticles to enhance OLED device efficiency [32]. Similarly, Goutam et al. investigated ZnO nanostructures, transitioning from zero-dimensional (0D) to three-dimensional (3D) configurations, as hole-injection layers (HILs) to evaluate their influence on device performance [33]. The incorporation of TiO_2_, whether in its anatase or rutile phases, significantly improves the performance of PEDOT/PSS in electronic applications. The behavior of hole polarons in TiO_2_ is particularly significant, influencing photocatalytic activity, charge transport, and recombination dynamics [23,34,35]. These exceptional properties position TiO_2_ as a vital component for enabling advanced functionalities in next-generation electronic devices.

**Figure 1:**
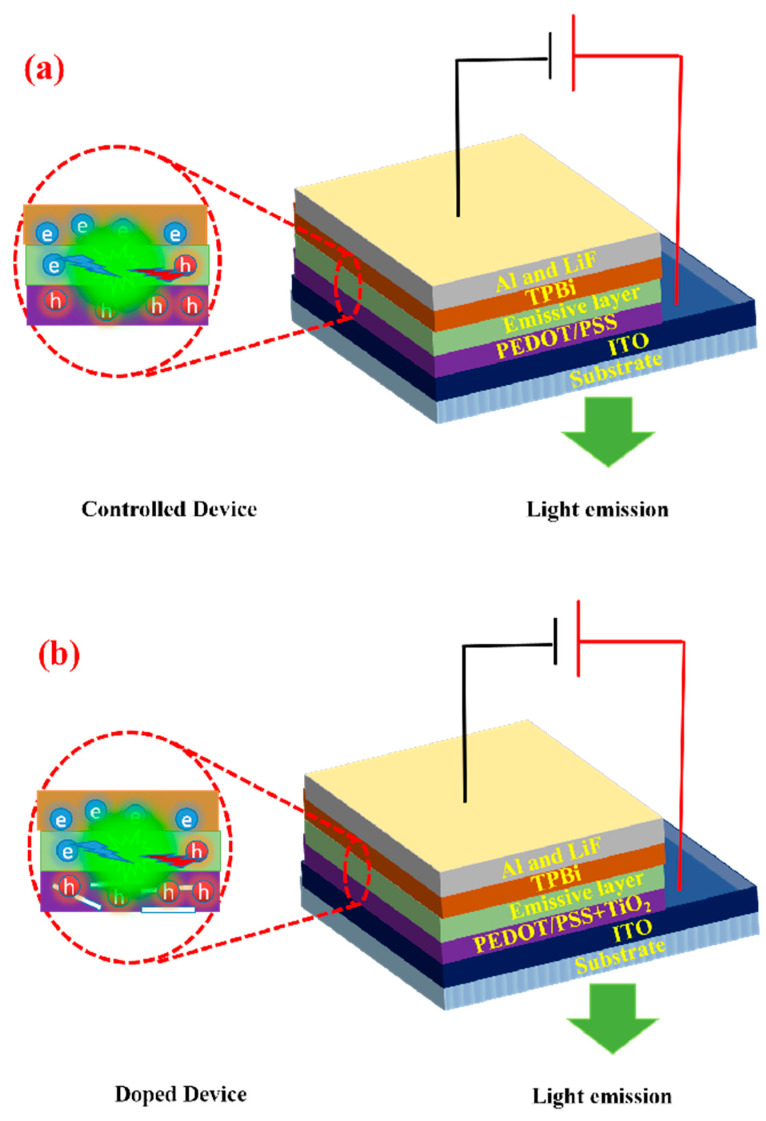
(**a**) Schematic diagram of controlled device; (**b**) schematic diagram of TiO_2_-NB-doped devices.

In this study, we explore the incorporation of TiO_2_ nanobelts (NBs) into PEDOT/PSS as nanocomposites to improve the efficiency of OLED devices. The TiO_2_ nanocomposites are designed to enhance charge transport pathways, improve hole-injection efficiency, minimize energy losses, and reduce charge scattering, thereby boosting the overall external quantum efficiency (EQE) and luminance of the OLEDs. By systematically optimizing the concentration and dispersion of TiO_2_ NBs in PEDOT/PSS, we aim to evaluate the impact of this compositional material on OLED performance metrics such as current density and voltage characteristics. This paper presents an in-depth investigation into the fabrication, characterization, and performance analysis of TiO_2_-NBs incorporated PEDOT/PSS nanocomposite layers in OLEDs; see Figure 1. Our findings provide a pathway for further material optimization and demonstrate the potential use of nanocomposite HILs in advancing OLED devices.

## 2. Results and Discussion

### 2.1. Morphological and Optical Properties

**Figure 2:**
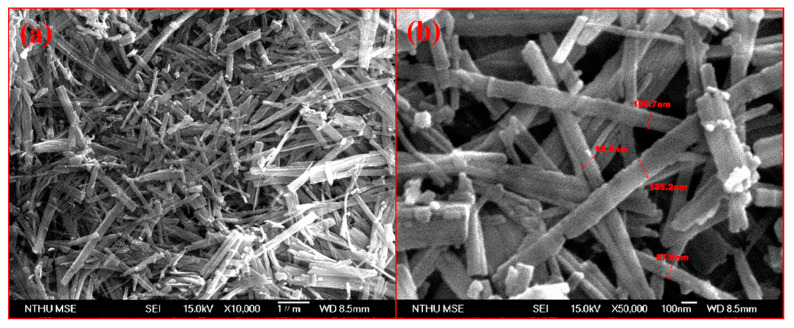
(**a**) SEM image at low magnification; (**b**) SEM image at high magnification of TiO_2_ NBs.

Scanning electron microscopy (SEM) analysis was performed to investigate the surface morphology of the TiO_2_ NBs. The SEM images, shown in Figure 2a,b, displayed that the nanobelts were randomly distributed, with an average width of approximately 90 nm. However, the nanobelts’ extremely small thickness posed challenges in obtaining high-resolution images with SEM, as the technique struggled to capture intricate morphological details accurately. These limitations necessitated the use of additional analytical methods to gain a deeper understanding of the structural characteristics of the nanobelts. To address this issue, transmission electron microscopy (TEM) was employed as a complementary technique, offering enhanced resolution and precision. TEM provided detailed characterization, including accurate measurements of nanobelt thickness, visualization of interplanar spacings, and insights into crystal structure and phase information. Furthermore, TEM allowed for a comprehensive assessment of the overall morphology, capturing fine structural features that could not be resolved through SEM alone. These aspects are discussed in greater detail in the TEM section, where the interplay between the structural and compositional attributes of the TiO_2_ NBs is elaborated upon. The SEM analysis highlights the significant potential of TiO_2_ NBs as HILs in organic light-emitting diode (OLED) devices. Their precisely defined dimensions and unique morphological properties provide a strong foundation for understanding their role in enhancing device performance. The ability to fine-tune the width, thickness, and overall morphology of these nanostructures opens avenues for optimizing the efficiency and functionality of OLED devices. This investigation underscores the critical relationship between the structural features of TiO_2_ NBs and their functional capabilities, contributing to advancements in OLED technology and broader applications in nanomaterial design.

**Figure 3:**
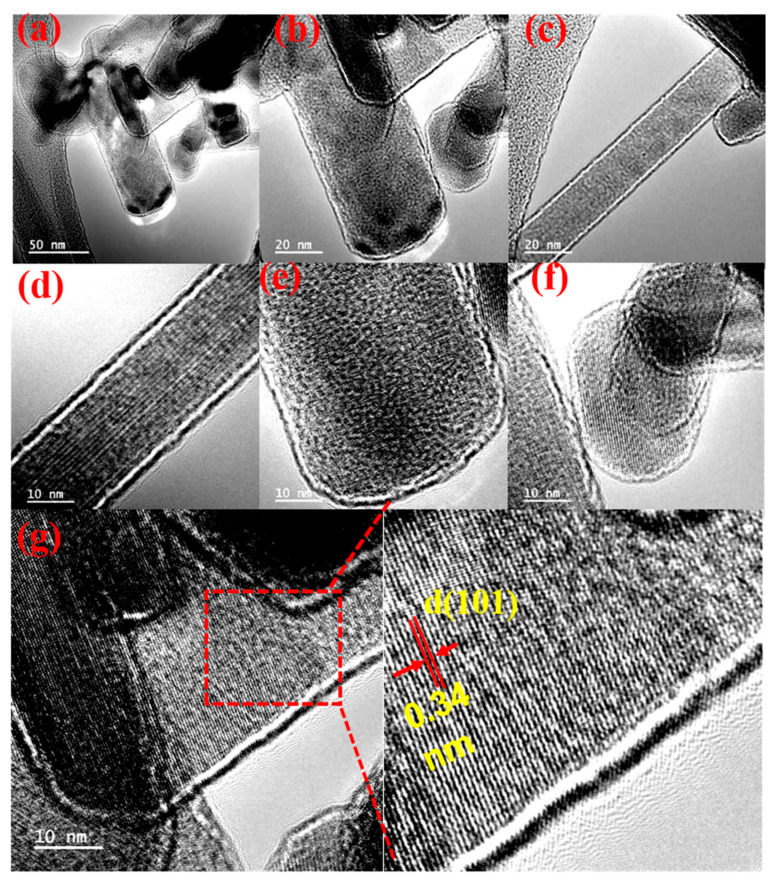
(**a**) TEM analysis of (**a**–**c**) at different resolutions of TiO_2_ NBs (**d**–**f**) at 10 nm resolution showing their line spacing of crystal growth; (**g**) HRTEM.

Transmission electron microscopy (TEM) was employed to investigate the structural and morphological characteristics of the synthesized TiO_2_ NBs. The TEM images, presented in Figure 3a,b, revealed the overall morphology of the nanobelts, which exhibited varying sizes with an average thickness of approximately 10 nm. For a clearer depiction of their length and width, Figure 3c highlights an individual TiO_2_. To provide a more detailed analysis, Figure 3d–f illustrate nanobelts with three distinct thicknesses, emphasizing the diversity within the synthesized samples. High-resolution TEM (HRTEM) imaging, as shown in Figure 3g, offered a magnified view of Figure 3f and revealed well-defined lattice fringes corresponding to a d(101) spacing of 0.34 nm. These lattice spacings are indicative of the crystalline structure of anatase-phase TiO_2_, with a preferred growth orientation along the [101] direction. This finding is further corroborated by X-ray diffraction (XRD) analysis, discussed in the XRD section. The HRTEM analysis provided critical insights into the structural details of the TiO_2_ NBs. The observed lattice fringes not only validated the crystalline nature of the material but also highlighted its high degree of structural uniformity. The anatase-phase confirmation, along with the preferred [101] growth orientation, underscores the material’s suitability for optoelectronic applications. Furthermore, the HRTEM imaging offered a nuanced understanding of the nanobelts’ thickness variations, providing valuable information on the synthesis precision and the material’s potential for targeted applications. To complement the structural analysis, energy-dispersive X-ray (EDX) spectroscopy was conducted to evaluate the elemental composition of the TiO_2_ NBs. The EDX maps, shown in Figure S3a, provided an overlapping distribution of titanium (Ti) and oxygen (O) atoms within the nanobelts, illustrating their uniform dispersion. Additionally, Figure S3b,c highlight the spatial distribution of Ti atoms using the Ti Kα1 emission line, while Figure S3c depicts the corresponding oxygen distribution through the O Kα1 emission. These findings confirmed the consistent and uniform distribution of Ti and O atoms, affirming the high purity and homogeneity of the synthesized TiO_2_ NBs. The integration of TEM, HRTEM, and EDX analyses provided a comprehensive understanding of the morphology, crystallinity, and elemental composition of the TiO_2_ NBs. The detailed characterization confirmed their structural uniformity, high purity, and consistent composition, thereby validating their potential for advanced applications in optoelectronic devices. This multi-faceted approach underscores the material’s robustness and adaptability, paving the way for its use in high-performance optoelectronic systems.

**Figure 4:**
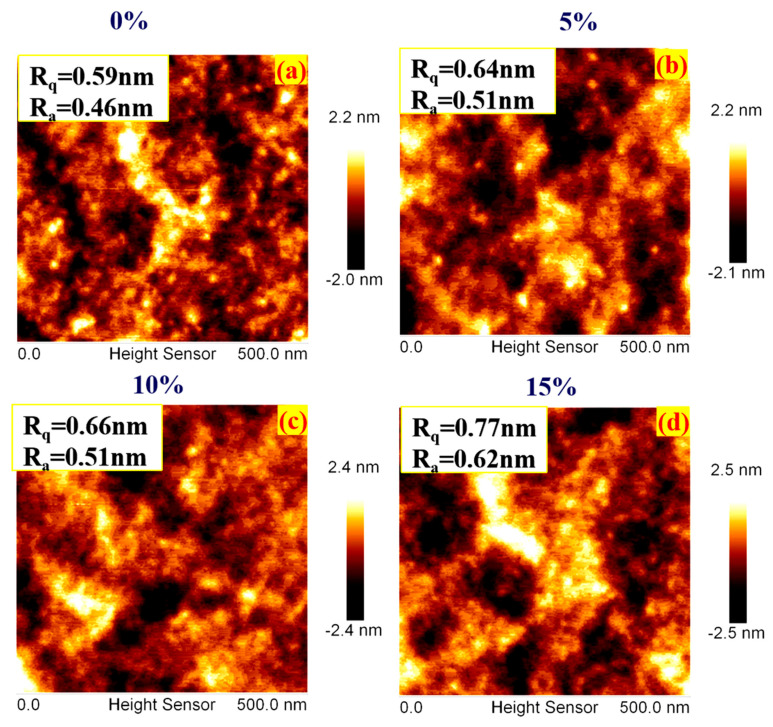
(**a**–**d**) AFM images. (**a**) PEDOT/PSS on ITO, (**b**) TiO_2_ NBs 5%, (**c**) TiO_2_ NBs 10%, (**d**) TiO_2_ NBs 15%, doped in PEDOT/PSS showing mean roughness of the samples.

Figure 4 illustrates the Atomic Force Microscopy (AFM) analysis of TiO_2_ NBs incorporated at varying weight percentages into PEDOT/PSS films, which were then spin-coated onto indium tin oxide (ITO) substrates. This analysis offers key insights into surface roughness and its potential impact on OLED device performance. Figure S2a shows the AFM scan of the bare ITO substrate, which displays moderate surface roughness, with an average roughness (Ra) of 0.73 nm and a root mean square roughness (Rq) of 0.94 nm, indicating a relatively uneven surface. Spin-coating PEDOT/PSS onto ITO, the surface roughness is significantly reduced, as evident in Figure 4a. The AFM images demonstrate a smoother surface, with the roughness metrics reduced to an Ra of 0.46 nm and an Rq of 0.59 nm. This decrease in surface roughness is attributed to the uniform morphology of the PEDOT/PSS layer, which effectively minimizes the irregularities of the underlying ITO substrate. However, when TiO_2_ NBs are doped into the PEDOT/PSS layer, a slight increase in surface roughness is observed. As shown in Figure 4b, the AFM analysis of the TiO_2_ NB-doped PEDOT/PSS film reveals an Ra value of 0.51 nm and an Rq value of 0.64 nm. Despite this marginal increase in roughness, the TiO_2_ NBs introduce a more intricate surface morphology, which can improve the interfacial contact between the hole-injection layer (HIL) and the emissive layer of the OLED. The enhanced interfacial contact resulting from the complex morphology of the TiO_2_ NBs is believed to facilitate more efficient charge injection at the HIL–emissive layer interface [36,37,38]. This improvement in charge injection efficiency is crucial for optimizing the overall performance of OLED devices [39,40]. While the surface roughness of the TiO_2_ NB-doped PEDOT/PSS layer is slightly higher than that of the pure PEDOT/PSS layer, this variation in roughness can play a pivotal role in enhancing interfacial properties, contributing to better charge transport and injection across layers. The AFM analysis illustrated in Figure 4a–d demonstrate that TiO_2_ NBs, despite introducing minor variations in surface roughness, may significantly enhance the interfacial characteristics and charge injection capabilities of OLEDs. These findings suggest that incorporating TiO_2_ NBs into PEDOT/PSS films can lead to improved OLED performance through optimized interfacial engineering [41].

X-ray diffraction (XRD) analysis was meticulously conducted on the synthesized TiO_2_ NBs to investigate their crystalline structure and verify their compositional purity. The resulting XRD pattern, shown in Figure S2, demonstrates that the nanobelts consist of both anatase and rutile phases, as evidenced by distinct diffraction peaks associated with specific lattice planes. For the anatase phase, characteristic peaks were observed at the following 2θ angles with their corresponding Miller indices: 25.3° (101), 37.8° (004), 48.0° (200), 53.9° (105), 55.1° (211), 62.7° (204), 68.8° (116), 70.3° (220), and 75.0° (215). Among these, the peak at 25.3°, corresponding to the (101) plane, was the most prominent, highlighting the highly crystalline nature of the anatase structure. These peaks closely match the reference JCPDS card number 21-1272 [42], thereby confirming the presence of the anatase phase along with contributions from the rutile phase. The observed peaks also align with anticipated lattice planes for both phases, underscoring the structural integrity and phase composition of the TiO_2_ NBs. Importantly, the absence of any extraneous or impurity-related peaks in the XRD pattern reaffirms the high purity of the synthesized nanobelts, reflecting the precision of the fabrication process. To further investigate the influence of thermal treatment, the XRD pattern of TiO_2_ NBs annealed at 450 °C was examined and is presented in Figure S2. This pattern revealed pronounced diffraction peaks corresponding to the (101), (002), and (200) planes, indicative of a well-ordered crystalline structure. The retention of both anatase and rutile phases under these annealing conditions demonstrates the structural and phase stability of the nanobelts. Notably, the anatase phase of TiO_2_ is known for its higher hole mobility [26], which is a beneficial property for its application as a hole-injection layer (HIL) in optoelectronic devices such as organic light-emitting diodes (OLEDs). This comprehensive XRD analysis highlights the successful synthesis of high-purity TiO_2_ NBs with a well-defined crystalline structure and a stable phase composition. The results confirm their structural integrity and underscore their potential utility in advanced optoelectronic applications, particularly where the unique properties of anatase TiO_2_ can enhance device performance.

**Figure 5:**
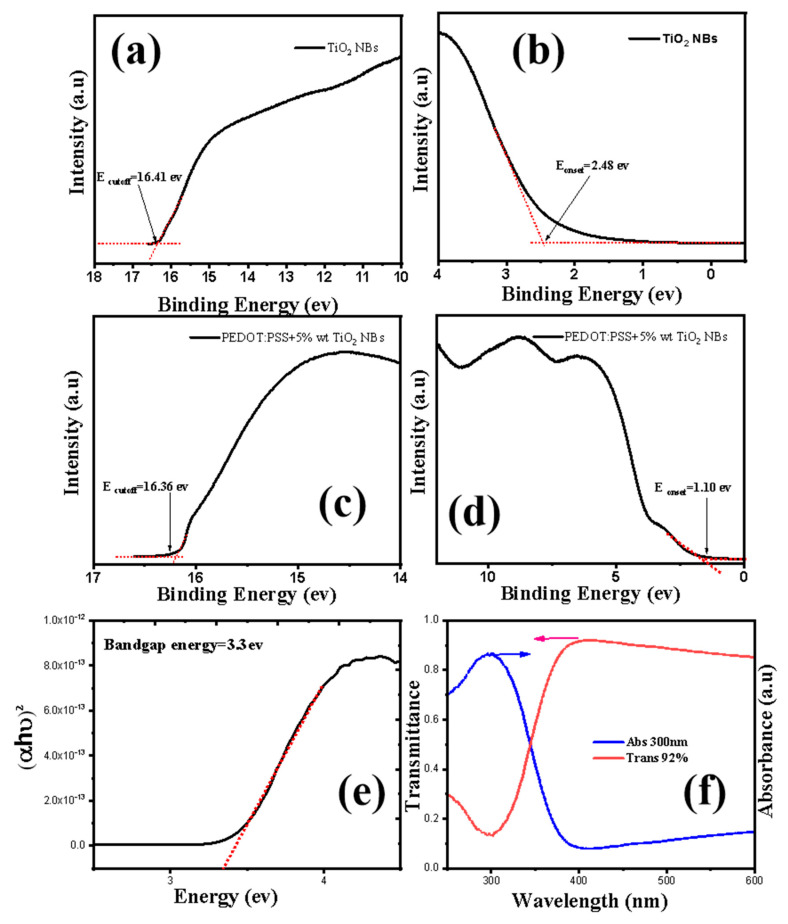
(**a**,**b**) showcase the UPS measurements of HOMO level of TiO_2_ NBs, and (**c**,**d**) represent the HOMO level of composite film. (**e**) UV analysis and (**f**) transmittance and absorbance of TiO_2_ NBs.

Ultraviolet–visible (UV–vis) spectroscopy was employed to examine the optoelectronic properties of the TiO_2_ NBs. The absorbance and transmittance spectra, shown in Figure 5a, reveal the behavior of TiO_2_ NBs, with a peak absorbance observed at 360 nm in the UV range and a high transmittance of 92% at this wavelength [43]. The optical properties of TiO_2_ NBs indicate their ability to absorb a significant fraction of light in the UV region while permitting a substantial portion of visible light to transmit through. This dual functionality makes them highly suitable for application in organic light-emitting diode (OLED) devices, where efficient light emission and minimal light obstruction are critical. The nanobelts exhibit an optical bandgap of approximately 3.37 eV, which was evaluated with the help of the Tauc plot method. The bandgap energy is extrapolated from the linear segment of the Tauc plot to the horizontal axis, providing an accurate estimation of the material’s bandgap [44]. The high transmittance of the synthesized TiO_2_ NBs in the visible spectrum is particularly advantageous for top-emitting OLED devices, as it facilitates efficient light outcoupling. Their UV absorption capability further enhances their functionality by potentially shielding underlying layers from UV damage while contributing to improved device stability. Consequently, these TiO_2_ NBs demonstrate strong potential as dopants in hole-injection layers (HILs), offering a synergistic combination of transparency, UV absorption, and energy-level alignment that can significantly enhance light emission and the overall performance of OLED devices.

Ultraviolet Photoelectron Spectroscopy (UPS) analysis is a powerful technique used to investigate the electronic properties of TiO_2_ NBs, particularly their energy levels and surface states. UPS enables the determination of the valence band maximum (VBM), work function, and surface electronic structure, which are critical for understanding and optimizing their performance in electronic and optoelectronic applications. For TiO_2_ NBs, UPS analysis typically reveals insights into their surface chemistry, band alignment with adjacent materials, and the influence of doping or surface modifications on their electronic states. The highest occupied molecular orbit (HOMO) of pure TiO_2_ NBs and the composite film of TiO_2_ NBs with PEDOT/PSS was determined using UPS. As depicted in Figure 5a,c, linear fitting of the left graph in the UPS analysis provided photoelectric emission cutoff energy (E_cutoff_) values of 16.41 eV and 16.36 eV for pure TiO_2_ NBs and composite TiO_2_ NBs, respectively. Similarly, linear fitting of the right graph in Figure 5b,d yielded optical emission onset energy (E_onset_) values of 2.48 eV and 1.10 eV for TiO_2_ NBs and composite TiO_2_ NBs, respectively [45]. The HOMO values for pure TiO_2_ NBs and the composite TiO_2_ film were calculated to be 7.27 eV and 5.9 eV, respectively [46]. The work function was determined from the left-hand graphs of Figure 5a,c, yielding values of 4.8 eV for pure TiO_2_ NBs and 4.9 eV for the composite film [47]. Furthermore, the LUMO energy level of the TiO_2_ NBs and composite films were estimated as 3.97 eV and 2.78 eV, respectively, using the relationship E_LUMO_ = −E_HOMO_ + Eg, where Eg, representing the optical bandgap, was determined through Tauc plot analysis. The corresponding optical bandgap for TiO_2_ NBs is 3.3 eV (Figure 5e) and for composite film is 3.1 eV (Figure S4). The UPS and UV analysis demonstrates that the calculated energy levels, including the HOMO, LUMO, and work function, exhibit a good alignment with the corresponding values of PEDOT/PSS and indium tin oxide (ITO). This close agreement indicates a compatibility between the TiO_2_ NBs and these widely used materials in optoelectronic applications. Such alignment highlights the potential of TiO_2_ NBs as effective dopants for the HIL, where they can play a critical role in optimizing charge transport pathways and enhancing charge injection efficiency. These properties make TiO_2_ NB-based composite films promising candidates for achieving superior performance in advanced optoelectronic devices [48].

### 2.2. Hole-Only Device and Hole Mobility Calculation

To calculate the hole mobility of the samples, we utilized the space charge limited current (SCLC) method. The SCLC of organic materials can be calculated by the Mott–Gurney (MG) equation, which is as follows. In hole-only devices, the current–voltage (J-V) characteristics typically feature two regions: the ohmic region and the space-charge-limited current (SCLC) region [49]. At low voltages, the current exhibits a linear relationship with voltage, indicative of ohmic behavior where charge injection is efficient, and the resistance is determined by the material’s intrinsic properties and electrode alignment. At higher voltages, the current transitions to the SCLC region, where it follows a quadratic dependence on voltage due to space-charge effects, reflecting the influence of charge carrier mobility, trap density, and active layer thickness. These regions provide critical insights into charge injection, transport mechanisms, and material optimization for OLED and other optoelectronic devices [50].

$$\mu =\frac{8J\;d^{3}}{9\varepsilon_{0}\varepsilon_{r}\nu^{2}}$$

where *μ* is the hole mobility, *J* is the current density, *d* is the thickness of the active layer, $\varepsilon_{r}$ is the relative permittivity (with a value of 3), $\varepsilon_{0}$ is the permittivity of free space (with a value of 8.85 × 10^−12^ F/m).

**Figure 6:**
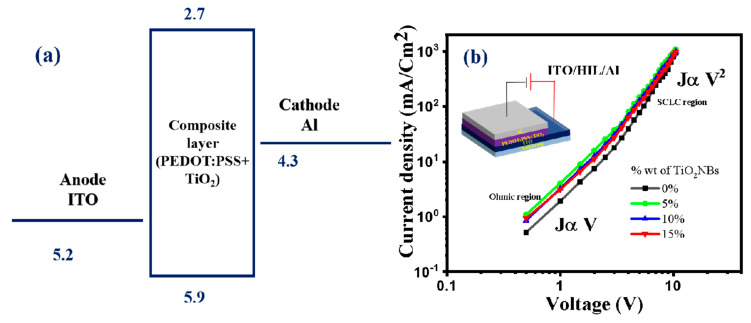
(**a**) Schematic diagram of hole-only device (PEDOT/PSS + TiO_2_ NBs); (**b**) current density vs. Voltage plot for determining hole mobility of HIL in doped and undoped devices (complete device structure is provided in the inset).

The fabrication of the hole-only device was carried out using the following structure: ITO as the anode and TiO_2_ NBs doped into PEDOT/PSS as HIL and Al as the cathode. A schematic energy-level diagram for the hole-only devices is presented in Figure 6a. The electrical characteristics of the hole-only device incorporating TiO_2_ NBs doped into PEDOT/PSS are detailed in Figure 6b. This comprehensive characterization underscores the suitability of TiO_2_ NBs for advanced applications in hole-injection layer and device optimization (Table 1).

### 2.3. Device Fabrication

**Figure 7:**
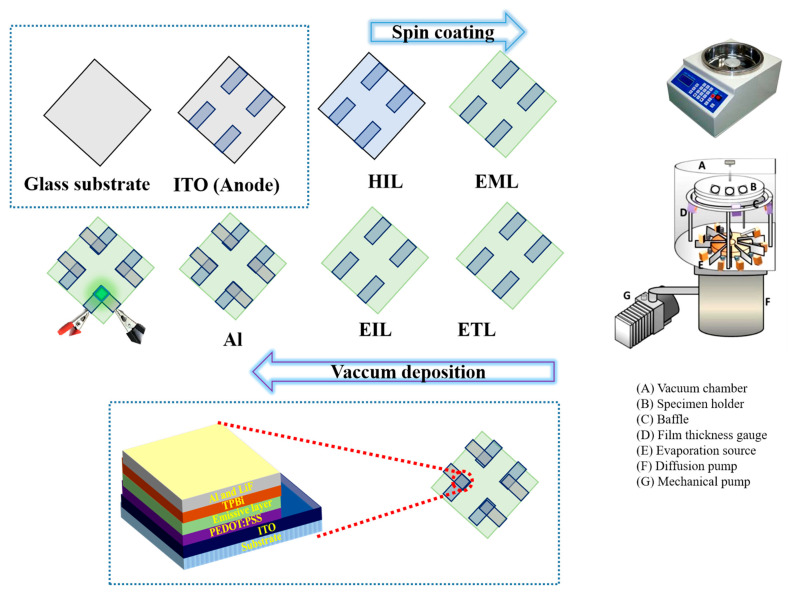
Fabrication process of solution process OLED.

A thorough, multi-step cleaning process was employed for pre-sputtered indium tin oxide (ITO) substrates with a sheet resistance of 25 Ω/sq. To begin, the substrates were treated in a soap solution to remove surface contaminants. They were then subjected to ultrasonic cleaning in two stages: first in acetone, followed by isopropyl butane, each for 40 min. The acetone stage was conducted at 50 °C, while the isopropyl butane stage was performed at 60 °C to ensure comprehensive removal of impurities. Following the ultrasonic cleaning steps, the substrates were placed in a pre-heated ultraviolet (UV) chamber for a 10 min UV treatment to eliminate any residual moisture, creating an optimal surface condition for subsequent layer depositions. Concurrently, the hole-injection layer (HIL) and emissive layer (EML) were prepared. The HIL was formulated by incorporating varying concentrations of TiO_2_ NBs, dissolved at a concentration of 1 mg/µL in deionized water, into a PEDOT matrix. In parallel, the EML was prepared by doping a 12.5 wt% concentration of Ir(ppy)_3_, a green phosphorescent emitter, into a TCTA host matrix. The hole-injection/transport and emissive layers were then spin-coated onto the substrates at 4000 rpm and 2500 rpm, respectively, with each coating step lasting 20 s. These processes took place in a nitrogen-filled glove box to prevent contamination from atmospheric oxygen or moisture. Once the spin-coating steps were completed, the substrates were transferred to a thermal evaporation chamber where the electron-transporting and electron-injection layers, along with an aluminum cathode, were deposited under a high vacuum of 10^−6^ torr. Throughout the evaporation process, a crystal sensor display was used to meticulously monitor the thicknesses of the layers and deposition rates, ensuring precision. Device performance was evaluated in an artificial dark room using a CS-100A system for current density–voltage–luminance measurements, and a Photo Research SpectraScan PR-655 spectrophotometer was employed to measure current efficacy, luminance, and power efficacy characteristics. Additionally, current–voltage characteristics were recorded with a Keithley 2400 voltmeter, and all measurements were conducted on devices with an active area of 0.09 cm^2^. A schematic representation of the OLED device fabrication process is provided in Figure 7.

### 2.4. Electroluminescent Properties

**Figure 8:**
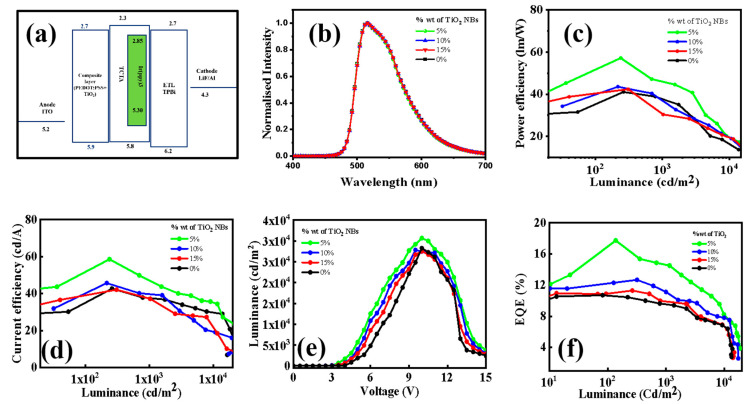
(**a**) Energy level diagram; (**b**) EL spectrum; (**c**) PE vs. luminance; (**d**) CE vs. luminance; (**e**) luminance vs. voltage; (**f**) EQE vs. luminance.

The thickness of the HIL in OLEDs plays a vital role in influencing the electroluminescence performance of the device. Achieving an optimal HIL thickness is essential for ensuring efficient charge transport, proper energy level alignment, and minimal recombination losses, all of which collectively enhance luminance, efficiency, and operational stability. If the HIL layer is too thin, it may result in incomplete coverage of the underlying layer, increased leakage currents, and poor hole injection, ultimately diminishing device performance. On the other hand, an overly thick HIL can lead to increased series resistance, thereby impeding charge transport and requiring higher operating voltages, which reduces power efficiency. To optimize device performance, particular attention was given to refining the thickness of the HIL during the fabrication process. The spin coater was meticulously calibrated to ensure consistent rotational speeds (rpm) across all devices, encompassing both doped and undoped configurations. The emissive layer in the device structure consists of Ir(ppy)_3_ serving as the dopant and TCTA functioning as the host material for the green phosphorescent OLED. The multilayer architecture of the OLED devices has the following structure: an ITO anode (150 nm), where the thickness of the HIL exhibits slight variation with the increasing doping concentration of TiO_2_ nanobelts, as shown in Figure S6; a TCTA/Ir(ppy)_3_ emissive layer (30 nm); a TPBi electron transport layer (40 nm); a LiF electron injection layer (1 nm); and an Al cathode (200 nm). All device fabrication procedures adhered to the methodology described in the corresponding section, with the process flow chart presented in Figure 7. Additionally, the energy-level diagram for the green OLED devices is illustrated in Figure 8a. This device design strategy aims to deliver OLEDs with superior performance characteristics, emphasizing eco-friendly processes and materials while maintaining cost-effectiveness.

For devices based on TiO_2_ NBs, different doping concentrations—0%, 5%, 10%, and 15%—of TiO_2_ NBs were incorporated into the PEDOT/PSS matrix. Of the tested devices, the one doped with 5% TiO_2_ exhibited the highest overall performance, achieving superior metrics in PE, CE, EQE, and luminance. Specifically, the TiO_2_ NB-based device, when operating at lower luminance levels, exhibited a PE_max_ of 57.2 lm/W, a CE_max_ of 58.6 cd/A, and an EQE_max_ of 17.7. As the luminance increased, a slight decrease in PE, CE, and EQE was observed (see Table S1). This result highlights that TiO_2_ NBs effectively mitigate the efficiency roll-off typically seen at higher luminance levels. In order to further evaluate the performance of the TiO_2_ NB-based devices, a control device OLED was fabricated. As illustrated in Figure 1a and detailed in Table S1, the control device displayed a PE_max_ of 41.6 lm/W, a CE_max_ of 42.7 cd/A, and an EQE_max_ of 10.4. Compared to the undoped devices, the TiO_2_ NB-doped devices achieved maximum enhancements of 39%, 37%, and 72% in PE, CE, and EQE, respectively. Determining whether hole injection or light extraction plays a more dominant role in enhancing OLED performance is challenging due to the intertwined contributions of both mechanisms. Efficient hole injection ensures balanced charge recombination, critical for optimal operation, while improved light extraction reduces optical losses, maximizing external quantum efficiency. In our study, XRD analysis confirmed the anatase phase of synthesized TiO_2_ nanobelts, known for excellent charge transport properties, with a strong (101) preferential orientation. HRTEM corroborated these findings, revealing a 0.34 nm interplanar spacing matching the (101) plane, highlighting the structural integrity and superior crystallographic alignment of the nanobelts. A hole-only device demonstrated that the composite material of TiO_2_ nanobelts with PEDOT/PSS exhibited higher hole mobility than PEDOT/PSS alone, attributed to efficient charge transport pathways enabled by TiO_2_. Additionally, the high refractive index of TiO_2_ mitigates refractive index mismatches [51], reducing total internal reflection and enhancing light extraction efficiency in OLEDs [52,53]. This dual enhancement underscores the synergistic role of TiO_2_ nanobelts in improving OLED performance. It was observed that both higher and lower doping concentrations of TiO_2_ NBs in PEDOT/PSS resulted in a decline in OLED performance. Several factors could account for this reduction in efficiency: (i) Lower Doping Concentration: At lower concentrations, the number of TiO_2_ NBs is insufficient for efficient charge injection and transport, which could result in reduced power, current, and quantum efficiencies [54,55]. (ii) Higher Doping Concentration: At higher concentrations, the increased density of TiO_2_ NBs within the PEDOT/PSS matrix could hinder light outcoupling and contribute to quenching, thus negatively impacting the device’s overall efficiency. Excessive doping may also promote molecular aggregation, resulting in the formation trapped state that dissipates energy as heat rather than light Moreover, high dopant concentrations can disrupt the charge transport properties of HIL by creating traps or scattering centers, causing imbalances in carrier injection and transportation, which further diminishes light output [56].

Therefore, optimizing the TiO_2_ NB doping concentration within the PEDOT/PSS matrix is essential to achieving the best possible device performance. Careful control of the doping levels is necessary to balance charge transport efficiency and light extraction, ensuring minimal performance degradation. Our findings demonstrate that the integration of TiO_2_ nanobelts into the PEDOT/PSS matrix serves a dual purpose: enhancing hole transport and improving light extraction. These synergistic effects contribute to the overall performance improvements observed in the OLED devices, illustrating the potential of this composite material for advancing next-generation optoelectronic technologies.

## 3. Conclusions

In conclusion, this study underscores the crucial role that TiO_2_ NBs play in enhancing the overall performance of organic light-emitting diodes (OLEDs). By doping TiO_2_ NBs within the PEDOT/PSS matrix, we explored their potential as HILs within OLED devices. The newly synthesized TiO_2_ NBs exhibited several advantageous properties, including a wide bandgap, moderate surface roughness, well-matched HOMO and LUMO levels, and excellent optical transparency. These characteristics collectively contributed to the significant improvements observed in device performance. The experimental results demonstrated a remarkable 39% increase in power efficacy (PE) and 37% improvement in current efficacy (CE), as well as a 72% enhancement in external quantum efficiency (EQE), when compared to devices without TiO_2_ doping. These improvements can be attributed to the effective charge transport and injection capabilities of the TiO_2_ NBs, as well as their ability to enhance light extraction and minimize efficiency roll-off at higher luminance levels. The findings from this study highlight the significant potential of TiO_2_ NBs in creating highly efficient OLED devices. The integration of TiO_2_ NBs into PEDOT/PSS has proven to be a promising strategy for improving OLED performance through enhanced charge injection and optimized optical properties. Moreover, the doping concentration of TiO_2_ NBs was found to be a critical factor in achieving these performance enhancements, as improper concentrations—either too high or too low—can negatively affect device efficiency. Future research endeavors may prioritize the further refinement of the dimension of TiO_2_ NBs while preserving their advantageous properties, such as high transparency and optimal energy levels. Efforts to minimize the size of these nanobelts hold the potential to significantly enhance their performance as HIL materials in OLED applications. Such advancements could contribute to the development of more efficient, cost-effective, and environmentally sustainable OLED devices. By addressing current size-related challenges and continually optimizing the material’s characteristics, we can propel the performance of OLED technology forward, enabling its broader adoption in displays, lighting, and a wide range of optoelectronic applications.

## Figures and Tables

**Table 1:** Hole mobility calculation of doped and undoped devices of hole-injection layer.

No.	Sample	*μ* (cm^2^/V·s)
1.	PEDOT/PSS	4.61 × 10^−4^
2.	PEDOT/PSS +5% TiO_2_	8.23 × 10^−4^
3.	PEDOT/PSS +10% TiO_2_	7.62 × 10^−4^
4.	PEDOT/PSS +15% TiO_2_	7.44 × 10^−4^

## Data Availability

Data is contained within the article and Supplementary Material.

## Supplementary Materials

The following supporting information can be downloaded at: https://www.mdpi.com/article/10.3390/nano15030199/s1, Figure S1. (a), 2d AFM image of ITO showing average roughness and (b), 3d topography of AFM image of TiO_2_ nanobelts. Figure S2. X-Ray diffraction of TiO_2_ nanobelts. Figure S3. EDX analysis of TiO_2_ nanobelts. (a) Bright-field image of two nanobelts with two different dimensions. (b) Distribution of O Kα1, indicating the presence of oxygen in the nanobelts. (c) Distribution of Ti Kα1, indicating the presence of Titanium in the nanobelts. Figure S4. UV analysis of composite film. Figure S5. Energy level diagram of HIL. Figure S6. Thickness of different HILs after doping TiO_2_ NBs into PEDOT:PSS. Figure S7. OLED photographs at varying voltage of the doped devices at 5.0 wt% doping concentrations. Table S1. A comprehensive list of the driving voltage, power efficacy, current efficacy, external quantum efficiency (EQE), CIE coordinates, and luminance of the OLED device based on TiO_2_ doped in the PEDOT:PSS matrix. 0% wt represent controlled device performance. Table S2. Demonstrates the device characteristics of OLEDs incorporating PEDOT:PSS doped with inorganic nanostructures, as reported in the literature. Refs. [32,33,57,58] are cited in the Supplementary Materials file.
