# Supplementary material for: TiO2-Nanobelt-Enhanced, Phosphorescent, Organic Light-Emitting Diodes
Source: Nanomaterials (Basel). 2025 Jan 27;15(3):199. doi: 10.3390/nano15030199 (PMC11821214; doi:10.3390/nano15030199)
Supplement: Supplementary file 1 [file nanomaterials-15-00199-s001.zip › nanomaterials-3413909-supplementary.pdf]

TiO_2 Nanobelts Enhanced Phosphorescent Organic Light-Emitting Diode

Sushanta Lenka[a], Shivam Gupta[b], Bushra Rehman[a], Deepak Kumar Dubey[c] , Hsuan Min Wang[a], Ankit Sharma[a], Jayachandran Jayakumar[a], Ching-Wu Wang[d] , *Nyan-Hwa Tai[b]* , Saulius Grigalevicius[e,]* , *Jwo-Huei Jou[a]**

[a,b]*Department of Materials Science and Engineering National Tsing Hua University 101, Sec. 2, Kuang-Fu Road, Hsinchu 30013, Taiwan.*

[c]*Advance Research, First Solar inc., Perrysburg, Ohio, USA, 43551.*

[d]*Graduate Institute of Optoelectronics, Department of Mechanical Engineering, National Chung Cheng University, Chiayi county 62102, Taiwan.*

[e]*Department of Polymer Chemistry and Technology, Kaunas University of Technology, Radvilenu Plentas 19, LT50254, Kaunas, Lithuania.*

Corresponding Authors:

S. Grigalevicius, Email: saulius.grigalevicius@ktu.lt

J.-H. Jou, Email: jjou@mx.nthu.edu.tw

Table of content

1. Experimental section

1.1. Instruments

1.2. Materials

1.3. Synthesis of TiO2 nanobelts (TiO_2 NBs)

2. **Characterizations**

1. Experimental section

1.1. Instruments

Ultraviolet-visible (UV-vis) absorbance measurements were performed using an HP-8453 diode array spectrometer. This instrument covers an extended spectral range spanning from 250 to 800

nm, which encompasses both the near-ultraviolet and visible regions. To obtain precise transmittance measurements, the same spectrometer was utilized, capturing data across the entire visible spectrum, ranging from 380 to 780 nm. These measurements enabled detailed insights into the optical properties of the samples under study.

For ultraviolet photoelectron spectroscopy (UPS) analysis, the U-3010 spectrometer was employed with a specific focus on characterizing the electronic properties of TiO_2 nanobelts. This analysis was crucial for determining the work function and probing the highest occupied molecular orbital (HOMO) levels, thereby providing important information on the electronic structure.

Scanning electron microscopy (SEM) imaging was conducted using two high-resolution instruments: the JEOL 6500F field-emission SEM (FESEM) and the Hitachi SU-8010. These devices facilitated the acquisition of detailed morphological data at the nanoscale level, revealing surface and structural characteristics. Furthermore, transmission electron microscopy (TEM) analysis was performed with a JEOL JEM F200, enabling high-resolution imaging of internal structures, which provided complementary information on the material's crystalline and morphological properties.

X-ray diffraction (XRD) analysis, essential for understanding the crystalline structure of the samples, was conducted using a Bruker D2 Phaser diffractometer equipped with Cu-Kα radiation (λ=1.54178 Å). This setup allowed the precise identification of crystallographic phases and lattice parameters.

Finally, atomic force microscopy (AFM) was carried out using the Bruker Dimension ICON instrument. This technique enabled the mapping of surface topography at the nanoscale, offering insights into surface roughness and other critical surface characteristics necessary for comprehensive material characterization..

1.2 Materials

All chemicals, which were of analytical grade, were directly used without any additional purification steps. Tris(4-carbazoyl-9-ylphenyl)amine (TCTA), 2,2',2"-(1,3,5-Benzinetriyl)-tris(1-phenyl-1-H-benzimidazole (TPBi), lithium fluoride (LiF), Tris(2-phenylpyridine)iridium ($Ir(ppy)_3$) were obtained from Shine Materials. Poly(3,4-ethylene dioxythiophene)-poly(stryenesulfonate) (PEDOT: PSS), sodium hydroxide (NaOH), sodium titanate hydrated ($Na_2Ti_3O_7.mH_2O$), hydrazine hydrate ($N_2H_4 \cdot H_2O$) were purchased from Sigma Aldrich and the filter paper was purchased from Merck Millipore.

1.3 Synthesis of TiO_2 nanobelts (TiO_2 NBs)

The hydrothermal method was utilized for the synthesis of TiO_2 nanobelts. Initially, 0.4 g of TiO_2 (≥99.5%, Aldrich) and 20 g of NaOH were dissolved in 50 mL of deionized (DI) water to form an aqueous solution. This mixture was mechanically stirred at room temperature for 2 hours, followed by ultrasonication for 15 minutes. The stirring and sonication cycle was repeated six times consecutively to ensure uniform dispersion. Subsequently, the resulting suspension was transferred into a 100 mL Teflon-lined stainless-steel autoclave and subjected to thermal treatment at 180 °C for 24 hours. Upon completion of the thermal process, the material was allowed to cool naturally to room temperature. The resulting white slurry, identified as sodium titanate ($Na_2Ti_3O_7$), was washed thoroughly with DI water and filtered until the pH of the washing solution reached 7. The neutralized slurry was then immersed in a 1 mol L^{-3} acetic acid (99.5%, DAEJUNG, Sihung, Republic of Korea) aqueous solution for 24 hours to convert the material into its protonated titanate form ($H_2Ti_3O_7$). The resulting $H_2Ti_3O_7$ was further washed with distilled water and filtered until the pH of the washing solution stabilized at neutral levels. Finally, the $H_2Ti_3O_7$ was dried at 80 °C for 24 hours and subsequently calcined at 450 °C for 2 hours to obtain the desired TiO_2 nanobelts.

2. Characterization

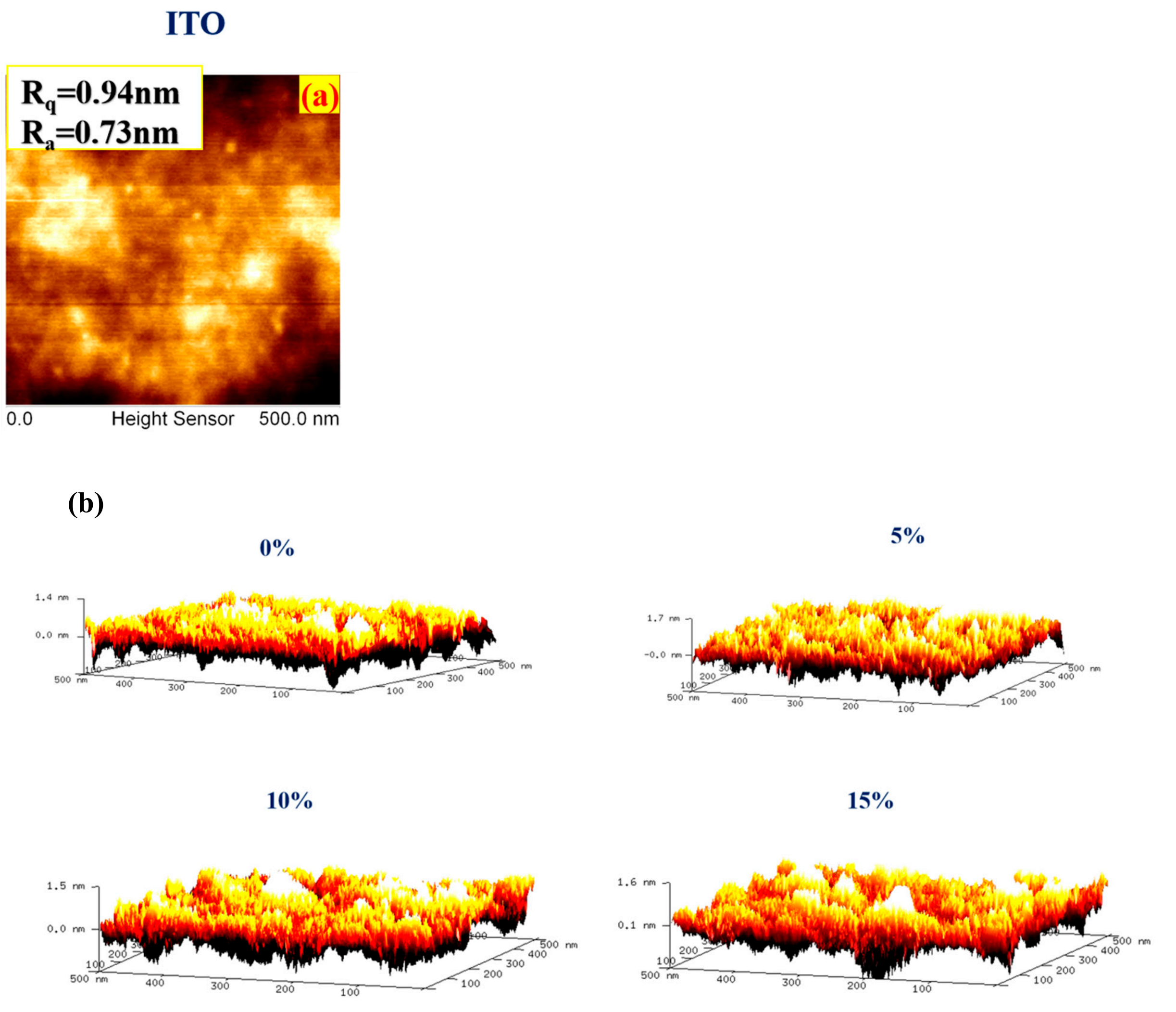

Figure S1. (a), 2d AFM image of ITO showing average roughness and (b), 3d topography of AFM image of TiO_2 nanobelts

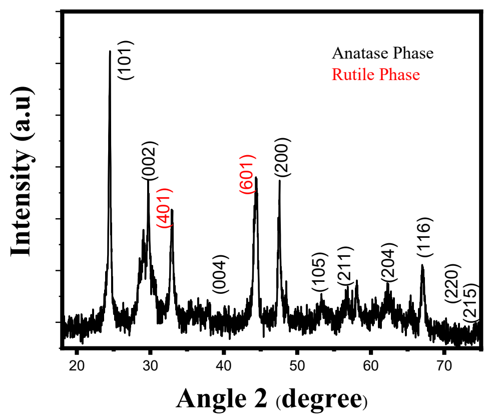

Figure S2. X-Ray diffraction of TiO_2 nanobelts

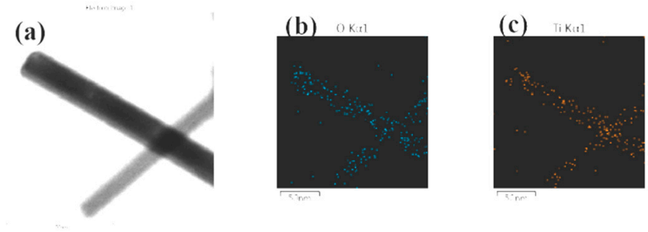

Figure S3. EDX analysis of TiO_2 nanobelts. (a) Bright-field image of two nanobelts with two different dimensions. (b) Distribution of O Kα1, indicating the presence of oxygen in the nanobelts. (c) Distribution of Ti Kα1, indicating the presence of Titanium in the nanobelts.

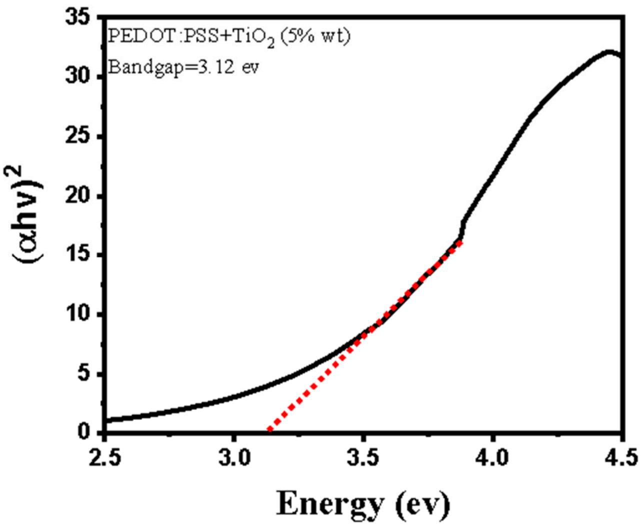

Figure S4. UV analysis of composite film.

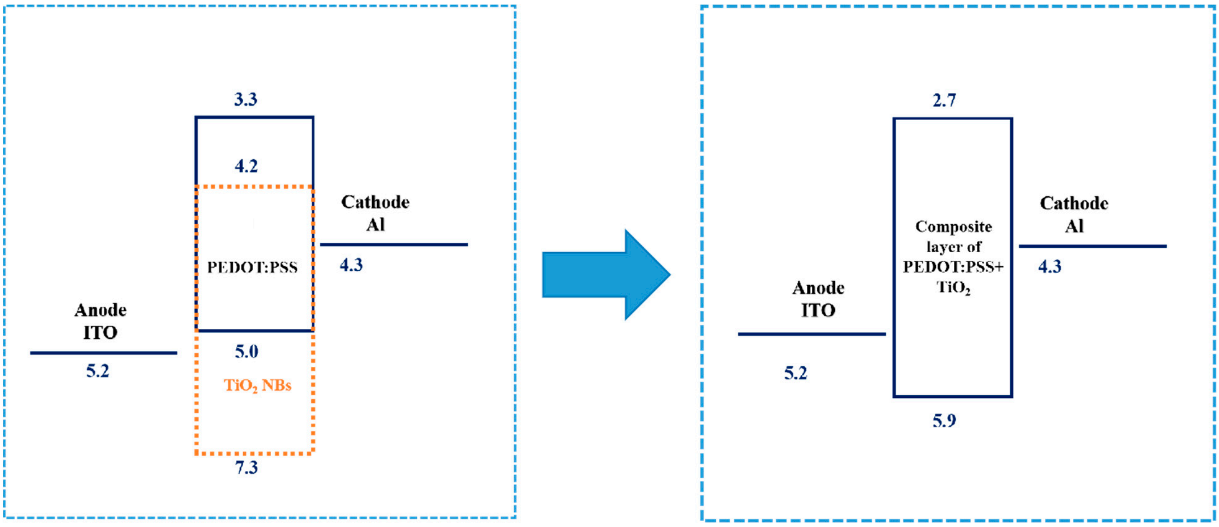

Figure S5. Energy level diagram of HIL .

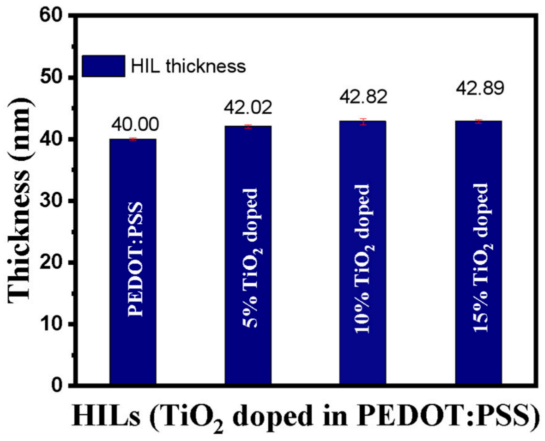

Figure S6. Thickness of different HILs after doping TiO_2 NBs into PEDOT:PSS.

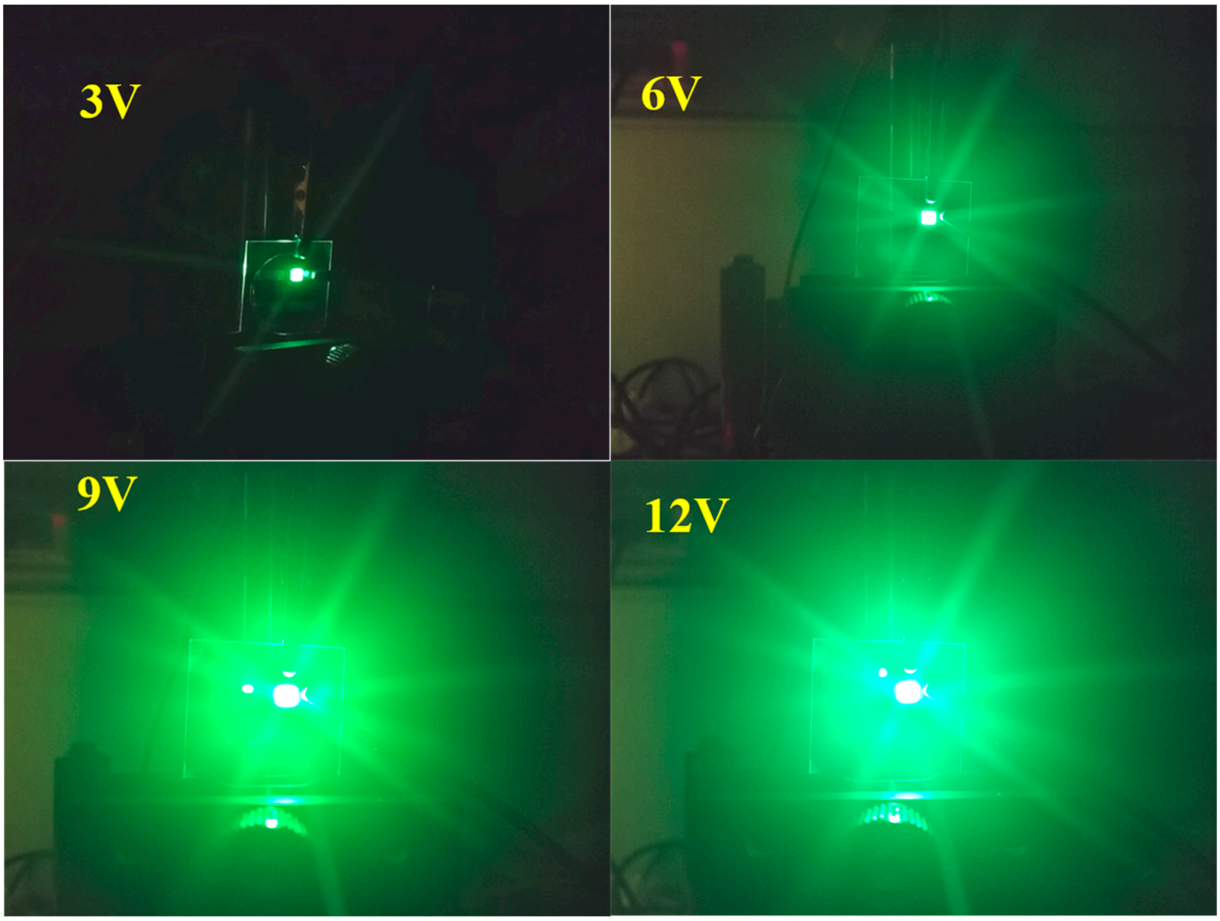

FigS7. OLED photographs at varying voltage of the doped devices at 5.0 wt% doping concentrations

Table S1. A comprehensive list of the driving voltage, power efficacy, current efficacy, external quantum efficiency (EQE), CIE coordinates, and luminance of the OLED device based on **TiO_2** doped in the PEDOT:PSS matrix. 0% wt represent controlled device performance.

%wt of TiO_2 NBs	Turn-on Voltage (V)	Operation Voltage (V)	Power Efficiency (lm/W)	Current Efficiency (cd/A)	EQE (%)	Voltage (Max. Luminance)	Max Luminance (cd/m^2)
		@100/1,000/10,000/max cd/m^2					
**0%**	**2.7**	**3.6/4.6/6.7**	**41.1/35.1/13.7**	**42.7/36.8/29**	**10.4/9.2/6.3**	**10**	**28350**
5%	2.6	3.1/4.0/5.8	57.2/44.6/18.7	58.6/43.8/34.4	17.7/14.5/7.2	10.5	30676
**10%**	**2.6**	**3.2/4.2/6.7**	**43.6/32.7/19**	**45.7/39.1/19.1**	**12.3/10.1/7.5**	**9.5**	**27840**
**15%**	**2.7**	**3.1/4.0/6.0**	**42.4/30.4/18.8**	**42.1/37.2/18.5**	**11.3/9.6/6.4**	**9.5**	**27530**

Table S2. Demonstrates the device characteristics of OLEDs incorporating PEDOT:PSS doped with inorganic nanostructures, as reported in the literature.

Materials	Function	Device performance	Remarks	References
ZnO NWs	HIL	49.5(PE),47.3(CE), 12.1 (EQE)	Improve hole mobilty	[1]
TiO_2 NP	HTL	PCE (8.9%)	Improved Conductivity and light transmittance	[2]
ZnO nanorod	HIL	11.3 (CE), 4.34 (PE)	Improved charge mobity	[3]
TiO_2 NPs	HIL	7.30 (CE), 3.15 (PE),	Improved light out-coupling	[4]
TiO_2 NBs	**HIL**	**57.2 (PE), 58.6 (CE), 17.7 (EQE)**	Improved hole mobility and light out-coupling	**This work**

References:

[1] P.Gautam , S. Gupta , I. Siddiqui , W.-Zhu Lin , D. Sharma , A. Ranjan , N.-Hwa Tai , M-Yen Lu , J-Huei Jou 0, 1, 2, and 3-Dimensional zinc oxides enabling high-efficiency OLEDs, Chemical Engineering Journal 495 (2024) 153220.

[2] Gaoyang Wang , Meng Zhang , Zhenye Li , Xingzhu Wang, Xiaomin Kang, Lei Ying : Efficient and stable organic solar cells enabled by incorporation of titanium dioxide doped PEDOT:PSS as hole transport layer: Progress in Organic Coatings Volume 183, October 2023, 107819

[3] N. Gupta, R. Grover , D. S. Mehta, K. Saxena , A simple technique for the fabrication of zinc oxide-PEDOT:PSS nanocomposite thin film for OLED application, synthetic metals , volume 221,November 2016.

[4] Nidhi Gupta, Rakhi Grover,Dalip Singh Mehta,Kanchan Saxena : Efficiency enhancement in blue organic light emitting diodes with a composite hole transport layer based on poly(ethylenedioxythiophene):poly(styrenesulfonate) doped with TiO2 nanoparticles:Displays Volume 39, October 2015, Pages 104-108
